# Beta-lactam combination treatment overcomes rifampicin resistance in *Mycobacterium tuberculosis*

**DOI:** 10.1007/s10096-025-05062-3

**Published:** 2025-03-06

**Authors:** Diana H. Quan, Trixie Wang, Elena Martinez, Hanna Y. Kim, Vitali Sintchenko, Warwick J. Britton, James A. Triccas, Jan-Willem Alffenaar

**Affiliations:** 1 Tuberculosis Research Program, Centenary Institute, The University of Sydney, Sydney, NSW 2006 Australia; 2 Centre for Infectious Diseases and Microbiology, The Westmead Institute, Westmead, NSW Australia; 3 School of Pharmacy, The University of Sydney, Sydney, NSW 2006 Australia; 4 Department of Clinical Immunology, Royal Prince Alfred Hospital, Sydney, NSW 2050 Australia; 5 School of Medical Sciences, Faculty of Medicine and Health, The University of Sydney, Camperdown, NSW 2006 Australia; 6 Sydney Institute for Infectious Diseases and the Charles Perkins Centre, The University of Sydney, Camperdown, NSW 2006 Australia; 7 Westmead Hospital, Westmead, NSW 2145 Australia

**Keywords:** Tuberculosis, Drug resistance, Beta-lactam, Beta-lactamase inhibitor

## Abstract

The significant global impact of tuberculosis (TB) on human health is exacerbated by the increasing prevalence of multi-drug resistant tuberculosis (MDR-TB) and the challenges of novel drug discovery for the treatment of drug-susceptible and drug-resistant strains of *M. tuberculosis*. Rifampicin is a key first-line TB drug and rifampicin resistance is a major obstacle to treating MDR-TB. Utilising existing antimicrobial drugs to supplement combination therapy and overcome rifampicin resistance is a promising solution due to their widespread availability and proven clinical safety profile. Therefore, this study aimed to explore the feasibility of using beta-lactam/beta-lactamase inhibitor combinations with rifampicin to inhibit the growth of multidrug-resistant *M. tuberculosis*. Based on inhibitory concentration (IC), oral bioavailability, pricing, commercial availability, five beta-lactams and the beta-lactamase inhibitor, clavulanate, were selected for testing. These were combined with rifampicin for in vitro testing against *Mycobacterium tuberculosis* H37Rv. Resazurin assays and colony forming unit (CFU) enumeration were used to quantify drug efficacy, Chou-Talalay calculations were performed to identify drug synergy and Chou-Martin calculations were performed to quantify drug dose reduction index (DRI). The combination of tebipenem-clavulanate/rifampicin and cephradine-clavulanate/rifampicin were found to be synergistic and highly effective against clinical isolates of MDR-TB, overcoming rifampicin resistance in vitro. Beta-lactam synergy may provide viable combination therapies with rifampicin to address the issue of drug resistance in TB.

## Introduction

Drug resistance in TB is categorised as multiple-drug resistant TB (MDR-TB), defined by resistance to rifampicin and isoniazid, and extensively drug-resistant tuberculosis (XDR-TB), defined as MDR-TB plus bacterial resistance to a fluoroquinolone and bedaquiline or linezolid. The burden of drug-resistance on healthcare systems has serious implications for the treatment and management of tuberculosis. Due to reliance on second-, third- and fourth-line antibiotics with limited efficacy and significant toxicity, treatment can cost over $1000 USD per patient and take up over 20 months of administration, support and counselling for adverse events [1]. As such, success rates for complete treatment of MDR-TB remain around 59% globally [1]. Only 19 of the 48 high-burden TB countries are not associated with significant rates of drug resistance, and 60% of all MDR-TB cases occur in some of the fastest-growing nations in the world, China, India, Russia, and South Africa [2]. Logistical barriers to the treatment of MDR-TB such as financial burden and treatment accessibility [3] must be taken into consideration when developing novel treatments, which should ideally prioritise cost, stability at room temperature and oral bioavailability.

However, the urgent need to identify novel drugs for the treatment of MDR-TB is countered by the high risks and exorbitant costs of antibiotic discovery. It was estimated in 2017 that the cost of developing an antibiotic is US$1.5 billion, while financial return was estimated at $46 million per annum [4]. As a result, several pharmaceutical companies have withdrawn from the field entirely [5]. While the preclinical phase of drug development has been termed the “valley of death” due to high rates of attrition and lack of progress towards clinical trial, there are several strategies which mitigate the risk of drug discovery and speed up clinical translation. One of these is the repurposing of pre-existing drugs.

A range of beta-lactam antimicrobial drugs have been tested against *Mycobacterium tuberculosis* with promising results [6]. As beta-lactam resistance is a well-established phenomenon dating back to 1929 [7], and the most effective mechanism for bacteria to counteract beta-lactam antibiotics is via the production of beta-lactamases [8], the addition of a beta-lactamase inhibitor such as clavulanate can reduce the incidence of drug-resistance to increase the utility of beta-lactam therapy for TB. Several beta-lactamase inhibitors have been tested and clavulanate was found to result in irreversible inhibition of the main M. tuberculosis beta-lactamase, BlaC [9, 10]. Amoxicillin/clavulanate has been found to be effective in patients with TB [11], and able to synergise with isoniazid, ethambutol and rifampicin for the treatment of drug-susceptible and -resistant isolates of *M. tuberculosis* [12]. Synergy between rifampin and faropenem, biapenem, doripenem, and meropenem for treatment of drug-susceptible and -resistant isolates of *M. tuberculosis* has also been reported [13]. Additionally, meropenem/clavulanate has been found to be effective against isolates of XDR-TB in vitro, as well as for the treatment of XDR-TB patients [14, 15], and the successful use of meropenem/clavulanate with linezolid for the treatment of XDR-TB has been established [13, 16, 17]. As beta-lactams and beta-lactamase inhibitors are orally well absorbed and cost-effective, it is vital to explore in detail the anti-TB efficacy of combinations of these drugs. Thus, the aim of our study is to characterise the drug interactions between existing beta-lactams and clavulanate with rifampicin, a key first-line drug for the treatment of TB.

## Materials and methods

### Bacterial strains, media and culture conditions

*M. tuberculosis* H37Rv (ATCC27294) was selected for this study as a commonly used virulent strain with high similarity to clinical isolates of *M. tuberculosis* [18, 19] and was used within three passages of seed vials of ATCC culture. The following clinical isolates were used for this study: *M. tuberculosis* Beijing 2847 (RIF/INH/EMB/PZA/AMK-resistant, isolated from patient lymph node), Beijing 3685 (RIF/INH/EMB/CIP/STR/ETH/KAN-resistant, isolated from patient sputum), Delhi 2994 (RIF/CIP-resistant, isolated from patient scalp aspirate), and Europe 5704 (RIF/INH/PZA/STR-resistant, isolated from patient sputum) isolates. All isolates were used within three passages of seed vials of original inoculum. Middlebrook 7H9 media supplemented with albumin-dextrose-catalase (ADC; 10% v/v), glycerol (0.2% v/v) and tyloxapol (0.05% v/v) was used to grow cultures which were incubated at 37 °C in a humidified 5% CO_2_ incubator.

### Screening for mycobacterial inhibition

Cephradine (Cayman Chemical), cefdinir (Cayman Chemical), tebipenem (Ambeed), cephalexin (Chemsupply), cefadroxil (Sigma-Aldrich), clavulanate (Cayman Chemical), and rifampicin (Sigma-Aldrich) were originally stocked in 100% DMSO.

Mycobacterial strains were grown to log phase and diluted to OD_600_ = 0.002. To formulate drug combinations a starting concentration of 3.1 pg/mL was used for rifampicin and 15 pg/mL for all beta-lactams. Two-fold dilutions of antibiotics were made in 7H9 media with a final concentration of 24 µg/mL clavulanate (if required) in 96 well plates. 100 µL mycobacterial suspension was added to each well containing 100µL of diluted antibiotics with clavulanate in triplicate, and incubated at 37°C for 7 days in a humidified incubator. Untreated controls did not receive antibiotics or clavulanate. Following incubation, 30 µL of resazurin (0.02% v/v in TDW) and 12.5 µL of Tween-80 (20% v/v in TDW) was added to each well and plates were incubated overnight at 37°C. Fluorescence readings were then taken at 590 nm after excitation at 544 nm using the FLUOstar Omega Microplate Reader (BMG Labtech, Ortenburg/Germany). Percentage viability was calculated in comparison to the average of untreated control wells after normalising for background readings. Z’-factors were greater than 0.5 for all assays. When testing against clinical isolates of drug-resistant *M. tuberculosis*, plates were incubated for 7 days before the addition of resazurin/Tween80 overnight. The results of the resazurin reduction assay were read by eye and minimum inhibitory concentration (IC) defined as the lowest drug concentration that inhibited bacterial growth. All experiments were performed in triplicate (i.e. three technical replicates) and repeated independently.

Cefadroxil and cephalexin produce fluorescent by-products which interfere with the resazurin reduction assay [20] and thus required colony forming unit (CFU) enumeration. For testing of cefadroxil and cephalexin, treatment of mycobacterial strains was conducted as described above, and aliquots of mycobacterial suspension were serially diluted ten-fold before plating on 7H10 agar. Agar plates were incubated 37 °C for 3 weeks in a humidified incubator before CFU counting.

### Combination analysis

Synergism between beta-lactams was determined by calculating the combination index (CI), CI = D1/Dx1 + D2/Dx2, where Dx1 and Dx2 indicate the individual dose of beta-lactams required to inhibit a given level of viability index, and D1and D2 are the doses of beta-lactams necessary to produce the same effect in combination, respectively [21]. CI values of < 1, = 1, and > 1 indicate synergism, additive effect and antagonism of the tested drugs, respectively. Additive drugs display an inhibitory effect equal to the sum of the individual effects of each drug. Antagonistic drugs display an inhibitory effect less than the sum of the individual effects of each drug [22]. The dose reduction index (DRI) for both beta-lactams were calculated using the multiple drug effect equation (DRI = Dx1/D1), where DRI values quantify how many folds of dose reduction result from drug combination in comparison to single drug treatment. To calculate CI and DRI, the percentage *M. tuberculosis* survival from the results of mycobacterial inhibition screening assays was converted to fraction affected (Fa) via complementation. Both CI and DRI were plotted against Fa and log-transformed to generate a Chou-Talalay and Chou-Martin plot providing visual illustration of synergism and dose-reduction [21].

## Results

### Synergistic activity of beta-lactams and clavulanate with RIF

**Fig. 1:**
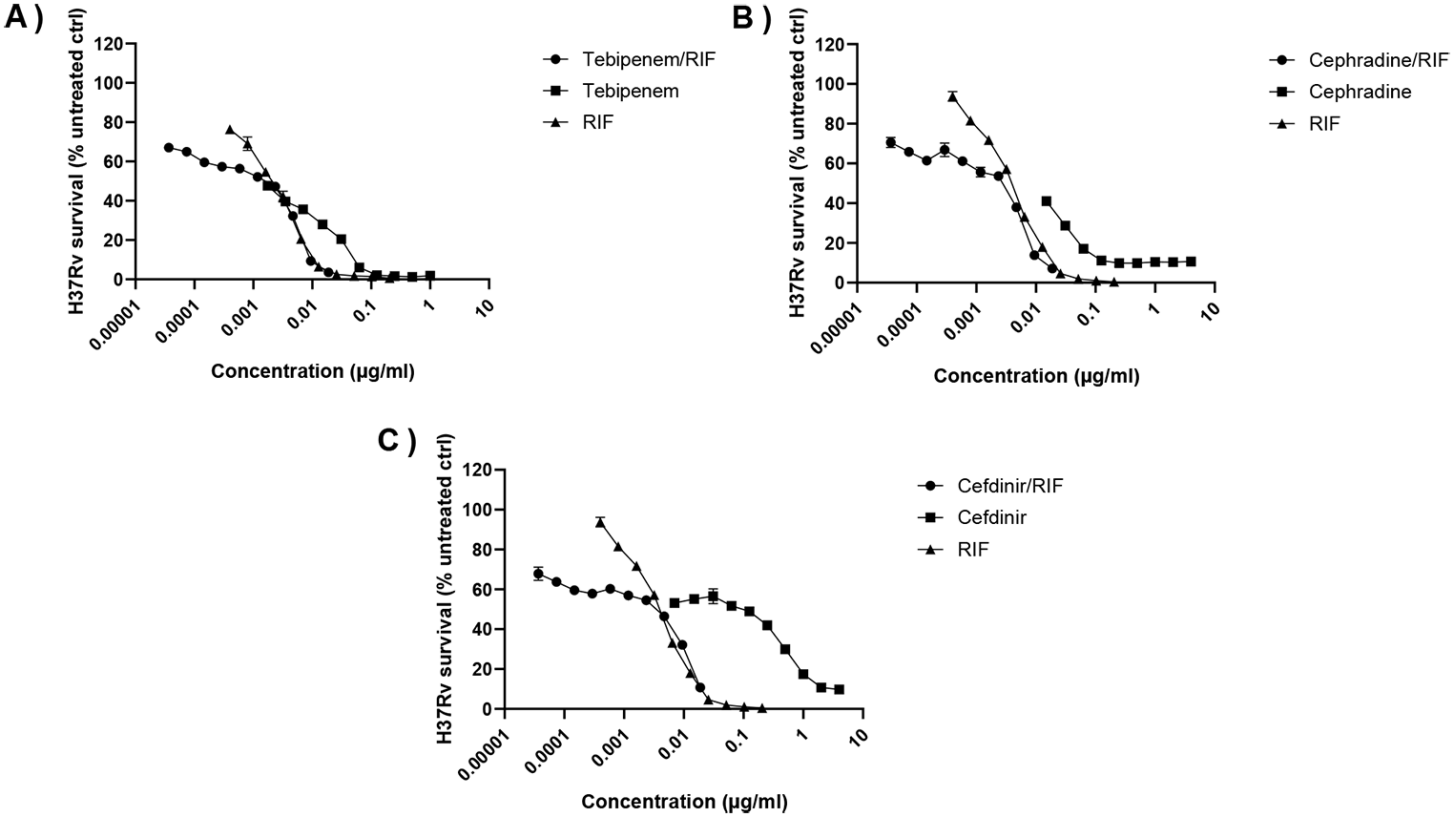
IC screening of beta-lactam and clavulanate combinations with RIF. (**a**) Tebipenem/RIF, (**b**) cephradine/RIF, and (**c**) cefdinir/RIF along with 24 µg/mL clavulanate were incubated with *M. tuberculosis* H37Rv (OD_600_ = 0.001) for five days before the addition of resazurin/Tween80, a further 24 h incubation period, and fluorescence reading at 590 nm. Graphs represent percentage viability of mycobacteria compared with untreated controls, and are representative of triplicate results from two independent experiments. Combination curves were plotted using added concentration of both antibiotics

**Fig. 2:**
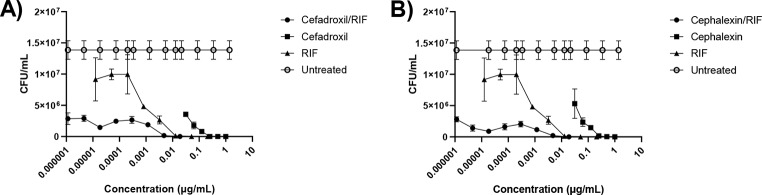
CFU screening of cefadroxil and cephalexin combinations with clavulanate and RIF. (**a**) cefadroxil/RIF, and (**b**) cephalexin/RIF along with 24 µg/mL clavulanate were incubated with *M. tuberculosis* H37Rv (OD_600_ = 0.001) for five days. Aliquots of mycobacterial suspension were serially diluted ten-fold before plating on 7H10 agar. Agar plates were incubated 37 °C for 3 weeks in a humidified incubator before CFU counting. Graphs represent triplicate results from two independent experiments

**Fig. 3:**
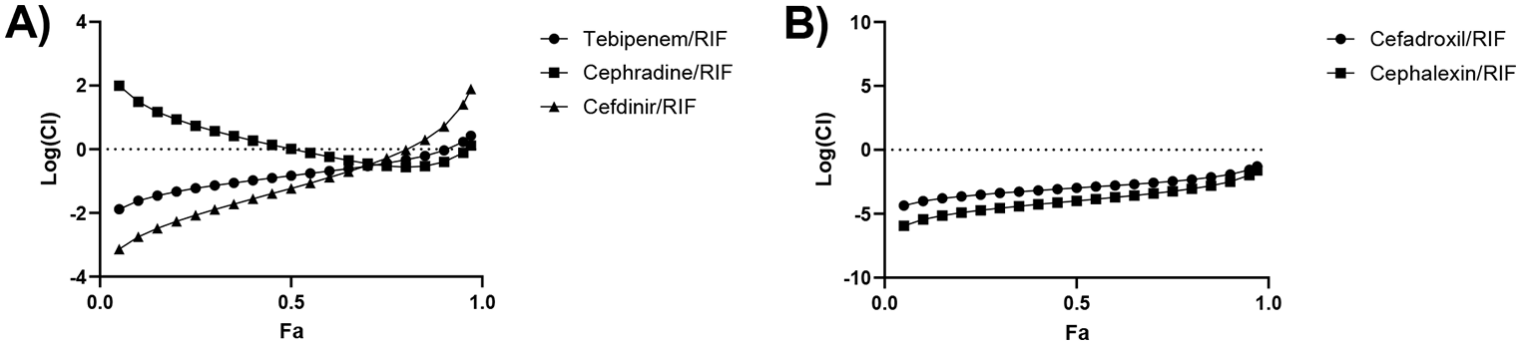
Evaluation of beta-lactam synergy with RIF. Serial two-fold dilutions of beta-lactam combinations were tested along with 24 µg/mL clavulanate over five days before the addition of resazurin/Tween80, a further 24 h incubation period, and fluorescence reading at 590 nm. (**a**) Chou-Talalay plot for tebipenem, cephradine and cefdinir combinations showing log(CI) against Fa, and (**b**) Chou-Talalay plot for cefadroxil and cephalexin combinations showing log(CI) against Fa. Graphs are representative of triplicate results from two independent experiments

To identify whether beta-lactams and clavulanate can be used effectively in combination with RIF for treatment of MDR-TB, tebipenem, cephradine and cefdinir were tested in combination with RIF, starting at suboptimal concentrations of 15 pg/mL for each beta-lactam and 3.1pg/mL for RIF (Fig. 1). To investigate cefadroxil and cephalexin synergy with RIF, combination testing of cefadroxil with RIF/clavulanate and cephalexin with RIF/clavulanate was performed via CFU enumeration (Fig. 2). Cefdinir/RIF with clavulanate was not synergistic for mycobacterial killing, with log(CI) values above 0 at Fa > 0.9 (Fig. 3). By contrast, the combinations of tebipenem/RIF and cephradine/RIF were found to be synergistic, with log(CI) values below 0 at Fa > 0.9, corresponding to beta-lactam concentrations > 9 pg/mL. In additional, cefadroxil/RIF and cephalexin/RIF were also found to be strongly synergistic, with log(CI) values below 0 at all Fa levels, corresponding to beta-lactam concentrations > 8 pg/mL. At Fa levels = 0.9, the log(CI) values for these combinations were − 0.04, -0.40, -1.92 and − 2.49 respectively (Fig. 3). Moreover, investigation of the dose reduction index (DRI) of the synergistic combinations revealed that combination therapy with beta-lactams and RIF greatly reduced the drug doses required to achieve IC_90_ inhibition (Table 1).

**Table 1:** Drug dose reduction index of synergistic beta-lactam and RIF drug combinations, in the presence of clavulanate, as determined using the multiple drug effect equation

Beta-lactam Combination	DRI of beta-lactam IC_90_	DRI of RIF IC_90_
Tebipenem/RIF	1.29	7.17
Cephradine/RIF	32.38	2.72
Cefadroxil/RIF	310.02	155.33
Cephalexin/RIF	1568.29	388.09
Cefdinir/RIF	2.80	0.01

### Antimycobacterial activity of beta-lactam combinations against clinical isolates of MDR-TB

To examine the utility of tebipenem/RIF and cephradine/RIF with clavulanate for the treatment of MDR-TB, this drug combination was tested against four clinical isolates of multidrug resistant *M. tuberculosis*, Beijing 2847, Beijing 3685, Delhi 2994 and Europe 5704. Combination therapy was able to reduce the IC required to inhibit the growth of both clinical isolates compared to treatment with tebipenem/clavulanate and cephradine/clavulanate alone (Table 2).

**Table 2:** Beta-lactam ICs^#^ of clinical MDR-TB isolates, with clavulanate, as determined via resazurin reduction assay. ^#^IC is defined as the lowest concentration that resulted in zero conversion of resazurin to resorufin, indicating zero mycobacterial growth

Clinical Isolate	Resistance Pattern	RIF IC (ng/mL)	Tebipenem IC (µg/mL)	Tebipenem/ RIF IC (µg/mL)	Cephradine IC (µg/mL)	Cephradine/RIF IC (µg/mL)
Beijing 2847	RIF/INH/EMB/PZA/AMK	> 0.1	0.125	0.018	0.030	0.001
Beijing 3685	RIF/INH/EMB/CIP/STR/ETH/KAN	> 0.1	0.015	0.001	0.125	0.018
Delhi 2994	RIF/CIP	> 0.1	0.004	0.001	0.004	0.001
Europe 5704	RIF/INH/PZA/STR	> 0.1	0.075	0.038	0.075	0.038

## Discussion

The global burden of MDR-TB continues to pose a significant public health challenge despite concerted efforts in tuberculosis control. The treatment outcomes for MDR-TB remain suboptimal, with cure rates typically below 60%, and a mortality rate that far exceeds that of drug-susceptible TB [1]. The current therapeutic regimen for MDR-TB, characterised by long durations (18–24 months) and the use of toxic second-line drugs, not only results in substantial adverse effects but also contributes to poor adherence and high rates of treatment discontinuation. As a consequence, there is an urgent and unmet need for more effective, safer, and shorter regimens.

Existing treatments for MDR-TB are often hampered by significant limitations, including drug resistance, the complexity of the regimen, and severe side effects such as ototoxicity, nephrotoxicity, and gastrointestinal disturbances. Moreover, the treatment regimen requires the use of injectable drugs, which are associated with poor patient compliance due to the need for prolonged hospital visits and monitoring. This situation is further compounded in low- and middle-income countries where access to quality care and adherence support is often limited. In this context, the development of more patient-friendly treatment protocols are crucial to reducing both the morbidity and mortality associated with MDR-TB. Research focusing on the discovery of new anti-TB compounds, repurposing existing drugs, and optimising treatment regimens through shorter and less toxic protocols is imperative.

This study investigated the known beta-lactams; cephradine, cefdinir, tebipenem, cefadroxil and cephalexin, and the beta-lactamase inhibitor, clavulanate, for potential synergistic activity against drug-susceptible and drug-resistant *M. tuberculosis*. Tebipenem, cephradine, cefadroxil and cephalexin were found to synergise with RIF (Fig. 3), and synergy was observed at low beta-lactam concentrations easily achievable in vivo [23–25]. Most significantly, the combination of tebipenem/RIF and cephradine/RIF with clavulanate was able to overcome rifampicin resistance in clinical isolates of MDR-TB (Table 2). This is consistent with a previous study of cephalosporins which reported strong synergy in cephalexin/RIF, cephradine/RIF and cefadroxil/RIF without clavulanate, and found that the activities of these combinations were both bactericidal and sterilising in vitro. However, when RIF was replaced by rifabutin, synergy was no longer observed [6]. The promising discovery that tebipenem/RIF and cephradine/RIF with clavulanate is effective against RIF-resistant clinical isolates of MDR-TB highlights a potential new approach to developing novel treatment regimens which can rapidly translate to clinical practice.

## Conclusion

Overall, the results of the current study support the use of beta-lactam combination therapy for the treatment of MDR-TB, and lay groundwork for future clinical trials.

## Data Availability

No datasets were generated or analysed during the current study.
